# Performance Analysis on Carrier Phase-Based Tightly-Coupled GPS/BDS/INS Integration in GNSS Degraded and Denied Environments

**DOI:** 10.3390/s150408685

**Published:** 2015-04-14

**Authors:** Houzeng Han, Jian Wang, Jinling Wang, Xinglong Tan

**Affiliations:** 1 School of Environment Science and Spatial Informatics, China University of Mining and Technology (CUMT), Xuzhou 221116, China; E-Mails: hanhouzeng@cumt.edu.cn (H.H.); tanxinglong@cumt.edu.cn (X.T.); 2 School of Civil and Environmental Engineering, University of New South Wales (UNSW), Sydney, NSW 2052, Australia; E-Mail: jinling.wang@unsw.edu.au

**Keywords:** carrier phase, GPS, BDS, MEMS IMU, tightly coupled, high accuracy positioning, ambiguity resolution, GNSS degraded and denied environments

## Abstract

The integration of Global Navigation Satellite Systems (GNSS) carrier phases with Inertial Navigation System (INS) measurements is essential to provide accurate and continuous position, velocity and attitude information, however it is necessary to fix ambiguities rapidly and reliably to obtain high accuracy navigation solutions. In this paper, we present the notion of combining the Global Positioning System (GPS), the BeiDou Navigation Satellite System (BDS) and low-cost micro-electro-mechanical sensors (MEMS) inertial systems for reliable navigation. An adaptive multipath factor-based tightly-coupled (TC) GPS/BDS/INS integration algorithm is presented and the overall performance of the integrated system is illustrated. A twenty seven states TC GPS/BDS/INS model is adopted with an extended Kalman filter (EKF), which is carried out by directly fusing ambiguity fixed double-difference (DD) carrier phase measurements with the INS predicted pseudoranges to estimate the error states. The INS-aided integer ambiguity resolution (AR) strategy is developed by using a dynamic model, a two-step estimation procedure is applied with adaptively estimated covariance matrix to further improve the AR performance. A field vehicular test was carried out to demonstrate the positioning performance of the combined system. The results show the TC GPS/BDS/INS system significantly improves the single-epoch AR reliability as compared to that of GPS/BDS-only or single satellite navigation system integrated strategy, especially for high cut-off elevations. The AR performance is also significantly improved for the combined system with adaptive covariance matrix in the presence of low elevation multipath related to the GNSS-only case. A total of fifteen simulated outage tests also show that the time to relock of the GPS/BDS signals is shortened, which improves the system availability. The results also indicate that TC integration system achieves a few centimeters accuracy in positioning based on the comparison analysis and covariance analysis, even in harsh environments (e.g., in urban canyons), thus we can see the advantage of positioning at high cut-off elevations that the combined GPS/BDS brings.

## 1. Introduction

Integration of GPS/INS is a popular tool for positioning due to their complementary error characteristics and has been widely studied for several decades [1]. The integrated system can be relied on to provide accurate and continuous position, velocity and attitude information in harsh environments. The overall performance of the integrated system heavily depends upon the availability and quality of GPS measurements which are used to calibrate inertial sensor errors [2]. The satellite-based positioning system can provide centimeter-level positioning accuracy by processing carrier phase measurements in differential mode, however it is a challenge to fix the integer ambiguities instantaneously with a long baseline (normally over 20 km) due to the fact atmospheric effects are hard to eliminate by double differencing, so it is popular to utilize the network real time kinematic (RTK) technique to cancel out the distance dependent error terms [3]. On the other hand, the GPS/INS integrated system has become a primary tool for the applications requiring high accuracy such as, e.g., Mobile Mapping System (MMS) and Airborne Mapping [4,5,6], which require rapid and accurate on-the-fly integer ambiguity resolution to achieve a few centimeters’ accuracy in positioning [7,8,9]. In order to improve the AR efficiency, the auxiliary measurements from INS are used to reduce the search space, which results in a significant improvement in ambiguity resolution in GPS-challenged areas [2,10], and the remaining GPS atmospheric errors can be estimated as additional unknowns in the integration system, thus extending the applicability of the GPS/INS integration system to long baseline navigation applications. However, the AR performance of kinematic positioning degrades significantly in constrained environments, such as unban environments with frequent signal blockage or when the low elevation multipath interference is significant, and the AR reliability relies highly on correct stochastic models for GNSS measurements, so it is not easy to model the GNSS observation noise, especially for kinematic applications as the vehicle maneuvers may affect the observation noise, resulting in inaccurate stochastic models for GPS and BDS combined measurements that inevitably result in biased solutions and deteriorated AR performance [11,12]. This contribution aims to improve the AR performance and associated algorithms to achieve more precise and reliable solutions. 

GPS can provide continuous, accurate navigation information in open-sky conditions with more than four satellites, however its accuracy and availability degrades significantly in the presence of signal blockage and multipath interference. To improve the GPS satellite availability, it is practical to add a new GNSS to the existing system. The Chinese BeiDou Navigation Satellite System came on-line to provide positioning, navigation and timing services in the Asia-Pacific region in December 2012 [13]. A lot of research has been conducted to evaluate the positioning performance of BeiDou, including relative positioning [14,15,16,17], precise point positioning [18,19] and orbit determination [20,21]. The obtained results are very promising and of importance to the development of the BDS. This research will integrate GPS, BDS and INS to improve the solution availability, accuracy and reliability. It is expected that AR performance will be significantly improved by the inclusion of BDS, thus the applicability of the integrated navigation system for seamless navigation will be increased.

The main limitation of INS is rapid navigation accuracy deterioration due to the uncompensated sensor errors, as the positioning performance for standalone INS is strongly dependent on the quality of inertial measurement unit (IMU) sensors [22]. In the past researchers have investigated the navigation performance of the high-end INS [23], but the use of high-end inertial sensors is limited due to their high prices. With the rapid progress in MEMS-based IMUs during the last few years, the sensor performance has been improved and the cost has been reduced substantially. The integration of GPS and MEMS IMUs has been widely and successfully applied in vehicular navigation applications [24,25,26,27]. This research will evaluate the bridging capability of MEMS-based INS and its enhancement in aiding GNSS ambiguity resolution.

The integration of GPS/BDS/INS can be implemented using a Kalman filter in either loosely, tightly or ultra-tightly mode. A tightly coupled integration scheme using GPS/BDS code and carrier phase measurements is implemented in this paper, which is considered to offer an advanced performance respect to the loosely coupled mode, the navigation states, sensor error states and other unknown parameters of interest are precisely estimated in the hybrid filter. In this contribution, single epoch AR performance and positioning accuracy of the GPS/BDS/INS combined system in kinematic positioning situation are evaluated. We first evaluate the effects of satellite availability improvement on AR efficiency and positioning accuracy by adding BDS. The successful and reliable AR requires precise float solutions, as bad precision of initial positions will degrade the AR performance, thus an INS aiding strategy is implemented which brings in a strong *a priori* constraint into the ambiguity search space to obtain a more reliable ambiguity fix. The improvement in resolving ambiguities that INS aiding brings to the combined system has not been evaluated in previous researches. A two-step ambiguity fixing strategy using wide-lane observations, L1 and L2 observations are applied sequentially to further improve the AR performance. In order to avoid the degradation that low-elevation multipath-contaminated observations brings to AR, an adaptive multipath factor-based modelling scheme is employed, which can lead to an improvement in the AR success rate.

The rest of this paper is organized as follows: in Section 2, the INS error model and measurement model for the TC GPS/BDS/INS integration system are briefly described. Section 3 presents the INS- aided integer ambiguity resolution method. In Section 4, the implementation of the proposed TC integration system is presented. Section 5 demonstrates the positioning performance of various integration configurations. The conclusions are presented in Section 6.

## 2. GPS/BeiDou/INS Tightly-Coupled KF Integration

### 2.1. INS Error Model

In land-based inertial navigation systems, the raw IMU data are processed using the strap-down algorithm, and the navigation errors grow with time in the absence of auxiliary measurements. In this research, the tightly coupled (TC) GNSS/INS integration architecture is implemented by adopting an extended Kalman filter (EKF), information from the blending filter is used to calibrate the INS sensor errors, the process model is derived from the psi-angle based INS error model [28]:

(1) $$\delta \dot{v}=-(2\omega_{ie}+\omega_{en})\times \delta v-\delta \psi \times f+\delta g+\nabla$$

(2) $$\delta \dot{r}=-\omega_{en}\times \delta r+\delta v$$

(3) $$\delta \dot{\psi }=-(\omega_{ie}+\omega_{en})\times \delta \psi +\varepsilon$$

where δ***r***, δ***v***, δψ are the position, velocity and attitude error vectors, respectively, ∇ and ε are the accelerometer error and gyro error vectors, respectively, δ***g*** is the gravity uncertainty error vector, ***f*** is the specific force vector, ω***_ie_*** is earth rotation vector, ω***_en_*** is the craft rate.

A twenty seven states INS error model is implemented here, which contains nine navigation error states that are expressed in the north-east-down (NED) navigation frame (three for position, three for velocity and three for orientation), six accelerometer and six gyro sensor errors (including three biases and three scale factors for each axis), three gravity uncertainty errors and three lever arm errors [4]. The detailed error states are given as follows:

$$\begin{array}{l} x_{Nav}\;={\left[\delta r_{N},\delta r_{E},\delta r_{D},\delta v_{N},\delta v_{E},\delta v_{D},\delta \psi_{N},\delta \psi_{E},\delta \psi_{D}\right]}^{T}\\ x_{Acc}\;={\left[\nabla_{bx},\nabla_{by},\nabla_{bz},\nabla_{fx},\nabla_{fy},\nabla_{fz}\right]}^{T}\\ x_{Gyro}={\left[\varepsilon_{bx},\varepsilon_{by},\varepsilon_{bz},\varepsilon_{fx},\varepsilon_{fy},\varepsilon_{fz}\right]}^{T}\\ x_{Grav}={\left[\delta g_{N},\delta g_{E},\delta g_{D}\right]}^{T}\\ x_{Ant}\;={\left[\delta L_{bx},\delta L_{by},\delta L_{bz}\right]}^{T} \end{array}$$

Accurate stochastic modeling of the MEMS-based INS bias is critical to improve the navigation performance. In this paper, the sensor errors of accelerometer and gyro on each axis are modeled as [29]:

(4) $$\nabla =\nabla_{b}+f_{b}\nabla_{f}+\eta_{a}$$

(5) $$\varepsilon =\varepsilon_{b}+\omega_{ib}^{b}\varepsilon_{f}+\eta_{g}$$

where η*_a_* and η*_g_* are white noise errors, $\omega_{ib}^{b}$ is the angular velocity. The cross-coupling errors and others are neglected for their small magnitude. The estimated bias terms are modeled as first-order Gauss-Markov (GM) processes which are a combination of turn-on bias and in-run bias, the scale factor errors are also modeled as first-order GM processes, described by the following equation [30]:

(6) $$\dot{x}=-\frac{1}{T_{c}}x+w$$

where *x* is random process with zero mean, *T_c_* is the correlation time and *w* is the driving noise, the stochastic model parameters are obtained using the Allan variance (AV) technique.

The gravity anomaly and deflections **x***_Grav_* are also modeled as first-order GM processes, while the leaver arm errors are treated as random constants. For long-range kinematic positioning applications, the double-difference ionospheric delays are added as additional unknown parameters which are modeled as a random walk.

### 2.2. Measurement Model

The measurement model of the relative positioning describes the relationship between the double-differenced (DD) measurements and the unknown parameters. In the TC GPS/BDS/INS filer, the DD pseudorange and carrier phase observation model can be described as:

(7) $$\Delta \nabla \rho^{*}=\Delta \nabla \rho_{0}^{*}+\Delta \nabla T^{*}+\Delta \nabla I^{*}+\Delta \nabla M^{*}+\Delta \nabla \varepsilon_{\rho }^{*}$$

(8) $$\lambda \Delta \nabla \phi^{*}=\Delta \nabla \rho_{0}^{*}+\Delta \nabla T^{*}-\Delta \nabla I^{*}+\lambda \Delta \nabla N^{*}+\Delta \nabla \varepsilon_{\phi }^{*}$$

where the Δ∇ notation refers to a double-differentiation, and “*” stands for “G” for GPS and “C” for BeiDou. **ρ** and ϕ denote the pseudo-range and carrier phase observations, the geometric distance between the receiver and satellite is **ρ**_0_, ***T*** is tropospheric delay, ***I*** is ionospheric delay, λ represents the carrier phase wavelength and ***M*** is the pseudorange multipath error, **ε**_ρ_ and **ε**_ϕ_ are the pseudo-range noise and carrier-phase noise.

In the TC system, the measurement model is linearized around the INS predicted position which is expressed in the Earth Centered Earth Fixed (ECEF) frame, and the design matrix in the linearized measurement model should multiply the direction cosine matrix $C_{n}^{e}$ to convert the position error states into the ECEF frame, and the same procedure should be conducted to convert lever arm error states from the body frame to the ECEF frame. As the above measurement model applies to short-range positioning applications, the tropospheric delay and ionospheric delay are assumed to be eliminated by double-differentiation, however for long distance kinematic positioning applications, the residual ionospheric uncertainties, which are not negligible, are estimated as unknown parameters by EKF.

For the combined GPS/BDS model, the system-specific double-differentiation is adopted for the frequency difference of the two systems, thus separated reference satellites are determined [17]. An accurate stochastic model for GNSS measurements can lead to very high precision positioning results. To account for the fact that observations at lower elevation angles suffer from more large atmospheric and multipath errors compared to the high elevation satellites, the *a priori* elevation-dependent weighting scheme is adopted in this research, which is described as:

(9) $$\sigma^{2}=\sigma_{0}^{2}/\sin^{2}(e)$$

where σ_0_ is standard deviation (STD) at zenith, and ***e*** is the elevation angle. The covariance matrix for the combined GPS/BDS system can be described as:

(10) $$R=\left[\begin{array}{cc} R_{GPS} & \\ & R_{BDS} \end{array}\right]$$

In the GPS/BDS/INS tightly-coupled system, an EKF is implemented for the non-linear system, the estimated INS sensor errors by the blending filter are then fed back to calibrate the accelerometer and gyro raw measurements. The solution of EKF is a recursive procedure which contains prediction step that is given by [31]:

(11) $$\left\{ \begin{array}{l} {\hat{x}}_{k}^{-}=f_{k-1}({\hat{x}}_{k-1}^{+})\\ P_{k}^{-}=\Phi_{k.k-1}P_{k-1}^{+}\Phi_{k,k-1}^{T}+Q_{k-1} \end{array}\right.$$

where ${\hat{x}}_{k-1}^{+}$ is the *a posteriori* state vector at epoch *k* − 1, ${\hat{x}}_{k}^{-}$ is the *a priori* state vector at epoch *k*, $\Phi_{k.k-1}$ is the state transition matrix from epoch *k* − 1 to *k*, and $P_{k}^{-}$ is the *a priori* covariance matrix of ${\hat{x}}_{k}^{-}$, $Q_{k-1}$ is the process noise matrix. The update step is provided as:

(12) $$\left\{ \begin{array}{l} K_{k}=P_{k}^{-}H_{k}^{T}{\left(H_{k}P_{k}^{-}H_{k}^{T}+R_{k}\right)}^{-1}\\ {\hat{x}}_{k}^{+}={\hat{x}}_{k}^{-}+K_{k}(z_{k}-h_{k}({\hat{x}}_{k}^{-}))\\ P_{k}^{+}=(I-K_{k}H_{k})P_{k}^{-} \end{array}\right.$$

where ***K***_*k*_ is the Kalman gain matrix, ${\hat{x}}_{k}^{+}$ is the *a posteriori* state vector at epoch *k*, and $P_{k}^{+}$ is the *a posteriori* covariance matrix of ${\hat{x}}_{k}^{+}$.

## 3. Integer Ambiguity Resolution for GPS/BeiDou/INS Integration

### 3.1. Integer Ambiguity Resolution Aided with INS

The precise kinematic positioning requires fixing the ambiguities fast and reliably. For a GNSS-only Kalman filter based integer ambiguity strategy that utilizes DD pseudorange and carrier-phase observables, the unknown parameters to be estimated are baseline vector and DD carrier-phase ambiguity vector, however the other navigation states and other unknown parameters in the TC integration filter are not included during the AR procedure. The state vector can be written as:

(13) $$X^{T}=\left[\begin{array}{cc} {X_{b}}^{T} & {X_{a}}^{T} \end{array}\right]$$

where ***X****_b_* is the unknown increments of the baseline vector and ***X**_a_* is the vector of double-differentiated carrier-phase ambiguities.

The corresponding variance-covariance matrix can be estimated by EKF:

(14) $$P_{k}^{+}={\left({\left(P_{k}^{-}\right)}^{-1}+H_{k}^{T}R_{k}^{-1}H_{k}\right)}^{-1}$$

(15) $${\left(P_{k}^{-}\right)}^{-1}={\left[\begin{array}{cc} p_{11} & p_{12}\\ p_{21} & p_{22} \end{array}\right]}^{-1}=\left[\begin{array}{cc} M_{11} & M_{12}\\ M_{21} & M_{22} \end{array}\right]$$

where the *a priori* covariance $P_{k}^{-}$ can be obtained from time update step of the TC integration filter (see Equation (11)), the initial ambiguity estimates and covariance are derived from the position predictions and the position covariance components.

On the other hand, the position vector derived from INS mechanization can be used as additional observables, the predicted pseudoranges $\Delta \nabla \rho_{0}$ are then computed with the aid of the INS predicted position ***r****_ins_*, then the EKF measurement model becomes:

(16) $$\underset{L}{\underset{︸}{\left[\begin{array}{c} \lambda \Delta \nabla \hat{\phi }-\Delta \nabla \rho_{0}\\ \Delta \nabla \hat{\rho }-\Delta \nabla \rho_{0}\\ 0_{3\times 1} \end{array}\right]}}=\underset{H}{\underset{︸}{\left[\begin{array}{cc} H_{b} & \lambda I_{n\times n}\\ H_{b} & 0_{n\times n}\\ I_{3\times 3} & 0_{3\times n} \end{array}\right]}}\underset{X}{\underset{︸}{\left[\begin{array}{c} X_{b}\\ X_{a} \end{array}\right]}}+\underset{\varepsilon }{\underset{︸}{\left[\begin{array}{c} \varepsilon_{\phi }\\ \varepsilon_{\rho }\\ \varepsilon_{ins} \end{array}\right]}}$$

where $\Delta \nabla \hat{\phi }$ and $\Delta \nabla \hat{\rho }$ represent carrier-phase and pseudorange measurements corrected with the predicted atmospheric delays, ***H****_b_* is the *n* × 3 DD design or geometry matrix, which contains receiver-satellite geometry information, *n* is the number of DD ambiguities.

Because the nominal user position is computed using the INS predicted position, then the virtual observations from INS can be represented as a zero vector. The strength of the measurement model is improved by adding INS measurements, so more precise estimates are expected to be obtained, and the integer ambiguity search space can be constrained to a certain amount.

As the INS position uncertainty is not correlated with GNSS observation noise, the second term on the right side of Equation (14) can be written as:

(17) $$\begin{array}{ll} H_{k}^{T}R_{k}^{-1}H_{k} & =\left[\begin{array}{ccc} H_{b}^{T} & H_{b}^{T} & I\\ \lambda I & 0 & 0 \end{array}\right]\left[\begin{array}{ccc} P_{\Delta \nabla \phi } & 0 & 0\\ 0 & P_{\Delta \nabla \rho } & 0\\ 0 & 0 & P_{ins} \end{array}\right]\left[\begin{array}{cc} H_{b} & \lambda I\\ H_{b} & 0\\ I & 0 \end{array}\right]\\ & =\left[\begin{array}{cc} N_{11} & N_{12}\\ N_{21} & N_{22} \end{array}\right] \end{array}$$

where $R^{-1}=P_{\Delta \nabla }$, and expand some terms to obtain the following expressions [2]:

$$\begin{array}{l} N_{11}=H_{b}^{T}P_{\Delta \nabla \phi }H_{b}+H_{b}^{T}P_{\Delta \nabla \rho }H_{b}+P_{ins}\\ N_{12}=\lambda H_{b}^{T}P_{\Delta \nabla \phi }\\ N_{21}=\lambda P_{\Delta \nabla \phi }H_{b}\\ N_{22}=\lambda^{2}P_{\Delta \nabla \phi } \end{array}$$

then the *a posteriori* observation matrix of the states can be re-written as:

(18) $$P_{k}^{+}=\left[\begin{array}{cc} P_{X_{b}} & P_{X_{b},X_{a}}\\ P_{X_{a},X_{b}} & P_{X_{a}} \end{array}\right]={\left(\left[\begin{array}{cc} M_{11} & M_{12}\\ M_{21} & M_{22} \end{array}\right]+\left[\begin{array}{cc} N_{11} & N_{12}\\ N_{21} & N_{22} \end{array}\right]\right)}^{-1}$$

Here we consider an EKF that without any prior knowledge of the states, that is to say ${\left(P_{k}^{-}\right)}^{-1}=0$, then Equation (14) is equivalent to a least square estimation problem, we can obtain:

(19) $$P_{X_{b}}^{0}={\left(H_{b}^{T}P_{\Delta \nabla \rho }H_{b}+P_{ins}\right)}^{-1}$$

(20) $$P_{X_{a}}^{0}=\frac{1}{\lambda^{2}}\left(H_{b}P_{X_{b}}^{0}H_{b}^{T}+R_{\Delta \nabla \phi }\right)$$

After estimating the real valued ambiguities ${\hat{X}}_{a}$ with the iterated EKF, the DD integer ambiguities are searched to satisfy the principle of integer least square (ILS) estimation as follows [32]:

(21) $${\overset{⌣}{X}}_{a}=\underset{X_{a}\in Z}{\text{argmin}}({\left(X_{a}-{\hat{X}}_{a}\right)}^{T}{P_{X_{a}}}^{-1}(X_{a}-{\hat{X}}_{a}))$$

The well-known LAMBDA and the conventional ratio-test with a fixed critical value are implemented in this study. The success rate of integer ambiguity estimation is related to ambiguity precision and correlation which is indicated by the ambiguity dilution of precision (ADOP), and ADOP, which we used to evaluate ambiguity resolution (AR) performance in the formal analysis, is denoted as [33]:

(22) $$\text{ADOP}={\sqrt{\det P_{{\hat{X}}_{a}}}}^{\frac{1}{n}}\;\;(\text{cycle})$$

The ADOP determines the size of the ambiguity search space, therefore the smaller ADOP indicates the faster and more reliable the ambiguity resolution process will be.

A detailed analysis of Equations (18)–(20) reveals the magnitude of $P_{{\hat{X}}_{a}}$ are functions of the following factors: (1) the prior knowledge of the states ($P_{k}^{-}$); by using the dynamic model, the position accuracies are further improved, which will be beneficial to ambiguity resolution; (2) the accuracy of the pseudorange measurements relative to the carrier-phase wavelength ($P_{\Delta \nabla \rho }$, λ); we can see the ambiguity success-rate is dependent on the measurement noise amplitude; (3) the satellite geometric strength (***H****_b_*); the augmentation of GPS with BeiDou and INS can significantly improve the model strength; (4) errors in position estimates derived from INS mechanization (***P_ins_***); the search space can be constrained to a certain amount by the introduced *a priori* position knowledge. For a GNSS-only least squares estimation approach, single epoch ambiguity resolution is difficult due to the insufficient precision of pseudorange measurements, and the multipath interference can be the dominant error source in the dynamic environment which makes it difficult to resolve ambiguities reliably. Specifically, when a GNSS signal outage occurs which is frequent in a typical urban environment, the performance of GNSS-only solution is limited. To maintain centimeter-level positioning performance in such constrained conditions, auxiliary measurements from INS are indispensable. If the INS can provide the position solution with sufficient accuracy over GNSS outage duration, the performance of carrier-phase ambiguities estimation is expected to be improved.

### 3.2. GPS/BeiDou Ambiguity Resolution Scheme

From the aforementioned analysis, the ambiguity resolution performance is affected by the accuracy of the pseudorange measurements relative to the carrier-phase wavelength. In this paper, linear combinations of original observables are formed to achieve wavelength amplification or noise reduction which is expected to improve AR performance.

Linear combination of pseudorange measurements yields Narrow-lane code combination:

(23) $$\begin{array}{c} \Delta \nabla {\hat{\rho }}_{\text{NL}}=(\frac{\Delta \nabla {\hat{\rho }}_{1}}{\lambda_{1}}+\frac{\Delta \nabla {\hat{\rho }}_{2}}{\lambda_{2}})\lambda_{\text{NL}}=\Delta \nabla \rho_{0}+\Delta \nabla T+\frac{f_{1}}{f_{2}}\Delta \nabla I\\ \;\;\;\;\;\;\;\;\;\;\;\;\;+\frac{\lambda_{\text{NL}}}{\lambda_{1}}(\Delta \nabla M_{1}+\Delta \nabla \varepsilon_{\rho_{1}})+\frac{\lambda_{\text{NL}}}{\lambda_{2}}(\Delta \nabla M_{2}+\Delta \nabla \varepsilon_{\rho_{2}}) \end{array}$$

where $\lambda_{\text{NL}}=\frac{c}{f_{1}+f_{2}}$ is called narrow lane, *c* is the speed of light, *f*_1_ and *f*_2_ are carrier frequencies, from Equation (23) we can infer that the STD of the multipath and observation noise on combination measurements is approximately 0.7 times those of L1 code measurements. On the other hand, by combining the carrier-phase measurements we can obtain Wide-lane combination:

(24) $$\begin{array}{c} \Delta \nabla {\hat{\Phi }}_{\text{WL}}=(\Delta \nabla {\hat{\phi }}_{1}-\Delta \nabla {\hat{\phi }}_{2})\lambda_{\text{WL}}=\Delta \nabla \rho_{0}+\Delta \nabla T+\frac{f_{1}}{f_{2}}\Delta \nabla I\\ \;\;\;\;\;\;\;\;\;\;\;+(N_{1}-N_{2})\lambda_{\text{WL}}+(\Delta \nabla \varepsilon_{\phi_{1}}/\lambda_{1}-\Delta \nabla \varepsilon_{\phi_{2}}/\lambda_{2})\lambda_{\text{WL}} \end{array}$$

with $\lambda_{WL}=\frac{c}{f_{1}-f_{2}}$ called wide lane.

The wide-lane ambiguity resolution becomes more easily compared to that of L1 or L2 for the longer wavelength, the wide-lane carrier-phase measurements can be used to facilitate original ambiguity resolution once the wide-lane ambiguities have been fixed, and then the accurate positioning solution can be obtained.

In the presence of strong multipath interference which can be expected in a harsh environment, realistic modeling of code covariance is critical, while the *a priori* elevation-dependent modeling scheme is insufficient to model the low-elevation multipath errors reasonably. To improve the efficiency of the variance model, a fading-memory scheme is implemented in this paper which accounts for time- and location-dependent multipath effects. The covariance matrix can be estimated on-line based on the predicted code residuals:

(25) $$V_{k+1}=(1-\beta_{k})V_{k}+\beta_{k}\cdot v_{k+1}v_{k+1}^{T}$$

(26) $$v_{k}={\left(\Delta \nabla \rho_{\text{NL}}-\Delta \nabla \rho_{0}\right)}_{k}$$

(27) $$\beta_{k}=(1-\alpha )/(1-\alpha^{k+1})$$

where ***V****_k_* is covariance matrix, ***v****_k_* is the residual series and α is the fading factor.

After the corresponding wide-lane ambiguities have been fixed, the similar residual-based adaptive procedure is employed to improve the reliability of Kalman filter, then Equation (26) becomes:

(28) $$v_{k}=\Delta \nabla \Phi_{\text{WL}}-\Delta \nabla \rho_{0}-(N_{1}-N_{2})\lambda_{\text{WL}}$$

The wide lane observations have the same variance-covariance structure with the original observations, while the difference is the noise level being increased by a factor of 5.74 compared to L1 measurements. Therefore an indirect covariance modeling strategy is employed for L1 measurements based on the wide lane stochastic models, and we expect to further improve the AR performance owing to the improved stochastic models.

## 4. Carrier Phase-Based Tightly-Coupled GPS/BDS/INS Integration Scheme

**Figure 1:**
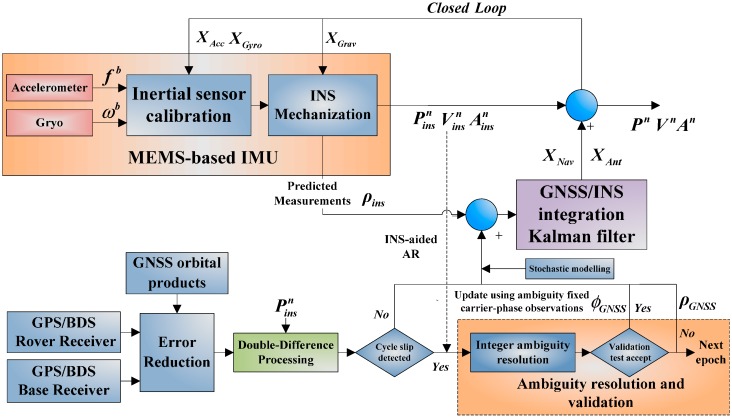
Implementation of the carrier phased-based tightly coupled GPS/BDS/INS integration.

The implementation scheme of the proposed carrier phase-based tightly coupled GPS/BDS/INS integration system is depicted in Figure 1. First, the INS is updated through mechanizaiton, and the double-differenced measurements are formed from GNSS obsevations of base station and rover station given the GNSS obital products and the predicted position output from INS mechnization, the double-differencing is implemented seperately for the individual system. A cycle slip detecion procedure then follows, the integer ambiguity resolution procedure is activated in the presence of cycle slip, the ambiguities are resolved with the aiding from INS, then a valitdation process is adopted to verify the fixed solutions. Then, the extended Kalman filter of the tightly-coupled integration directly fuses the ambiguity fixed DD carrier phase observarions with the INS predicted pseudoranges to estimate the error states, whereas the DD pseudorange measurements should be used when the GNSS AR validation failed. Finally, the navigation solution of INS are then calibrated with the estimated navigation error states (*i.e.*, the position, velocity, attitude and the lever arm offset), the estimated inertial sensor error states (bias and scale factor) are fed back to correct the raw INS measurements, the gravity errors are also fed back to INS mechanization. It is expected that the integation system can be relied on to provide accurate, continuous performance in harsh environments (e.g., urban canyons or low-elevation multipath dominant environments).

**Figure 2:**
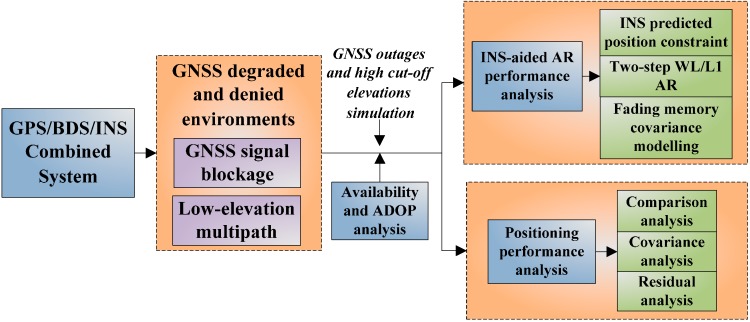
Performance analysis of the GPS/BDS/INS combined system in GNSS degraded and denied environments.

The Chinese BDS became operational in the Asia-Pacific area since 27 December 2012, and the new system will enhance the satellite visibility and availability. It is important to understand the capabilities of combining GPS, BDS and MEMS-based INS for high accuracy positioning applications, and the benefit of adding BDS data is considerable relative to the GPS-only case, specifically for vehicular navigation applications in constrained environments. In this paper, we are trying to assess the AR performance of and positioning performance of the GPS/BDS/INS combined system under deteriorated circumstances. The analysis is carried out for different cut-off elevation situations, ranging from 10° to 40°. As part of this, INS-aided AR performance analysis is done, the role of INS predicted position constraints, as well as adaptive fading memory stochastic modeling are evaluated given the improvement in ambiguity fixing rate, both the fixing rate of GNSS-only system and combined system are provided for comparison. The INS bridging capability is evaluated by introducing GNSS outages with different durations, the adding information from INS is still beneficial for fast ambiguity recovery after the GNSS signal is relocked. The positioning performance of the combined system which is affected by different contributing factors is evaluated by conducting comparison analysis, covariance analysis and residual analysis. The overall flowchart of performance analysis is provided in Figure 2.

## 5. Results and Discussion

**Figure 3:**
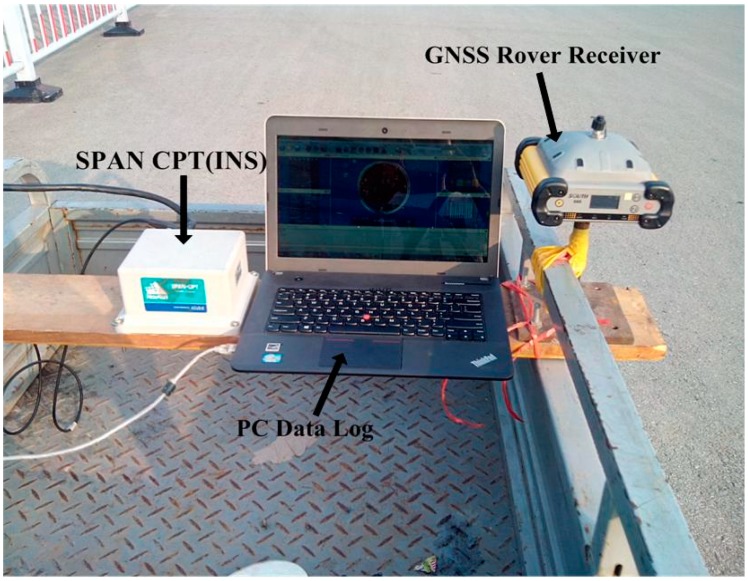
Testing platform.

A field vehicular test was carried out to evaluate the performance of the developed GPS/BDS/MEMS-based tightly coupled system. The test area is a typical area characterized by foliage and a lake, so the satellite positioning performance is limited because of signal blocking and multipath problems. The integrated navigation system consists of a SPAN-CPT system, which is an integrated navigation system consisting of a MEMS-based IMU (whose specifications are shown in Table 1) and a NovAtel OEM4 receiver, and a dual-frequency SOUTH GPS/BDS receiver fixed on a vehicle driving at a low speed (Figure 3). Another same receiver set up as the reference station was mounted on the roof of the School of Environmental Science and Spatial Informatics (SESSI) building, on the campus of China University of Mining and Technology (CUMT), Xuzhou, Jiangsu, China, the baseline separation was less than 5 km.

**Table 1:** IMU sensor specifications.

Gyro		Accelerometer	
Bias Offset	±20°/h	Bias Offset	±50 mg
Bias Repeatability	±3°/h	Bias Repeatability	±0.75 mg
In Run Stability	1°/h (1σ)	In Run Stability	0.25 mg (1σ)
Scale Factor Stability	1500 ppm	Scale Factor Stability	4000 ppm
Angular Random Walk	0.0667°/√h	Velocity Random Walk	55 mg/√Hz

**Figure 4:**
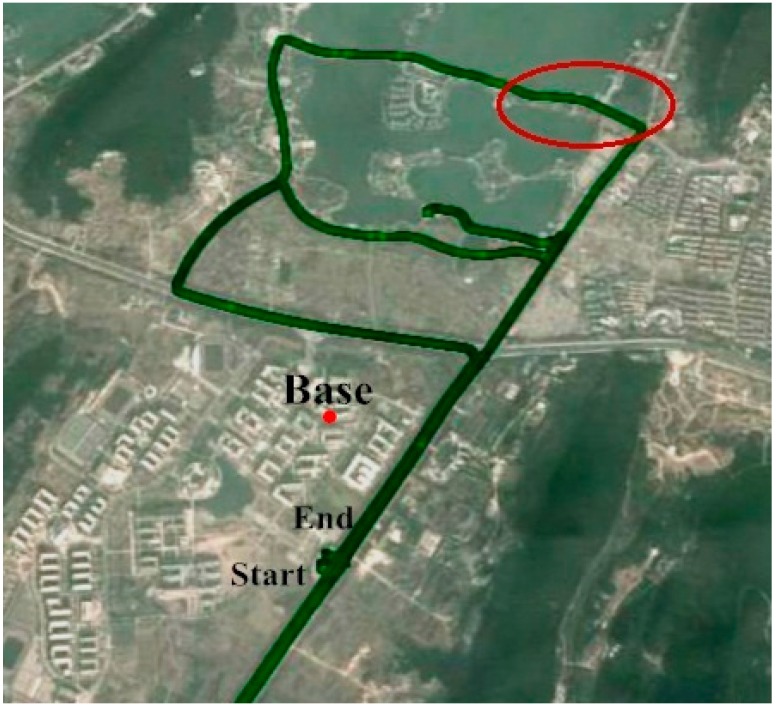
Field test trajectory (vehicle operated under foliage in the area marked by a red circle).

**Figure 5:**
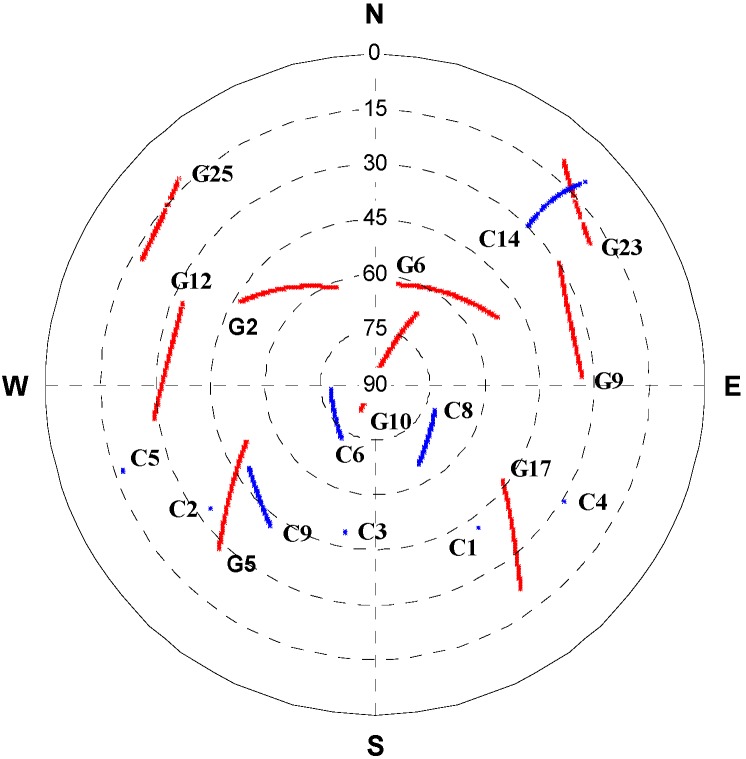
Sky plots of GPS/BeiDou satellites during the field test.

The raw carrier phase and pseudorange data were collected at a 1 Hz rate, the IMU raw measurements were recorded at a sampling rate of 100 Hz. The test duration was approximately 72 min and the test trajectory is shown in Figure 4. Figure 5 shows satellite positions in the sky relative to current vehicle position. Figure 4 shows nine BeiDou satellites were visible during the test, including five GEO satellites (PRN 1, 2, 3, 4, 5), three IGSO satellites (PRN 6, 8, 9) and one MEO satellite (PRN 14), while three other MEO satellites were invisible during the navigation period. It is obvious the GNSS solution availability is improved by including the BeiDou observations, especially in harsh environments; the positioning performance is degraded for standalone GPS due to the weak satellite geometry configuration when the vehicle operated under foliage (marked by a red circle in Figure 4).

**Figure 6:**
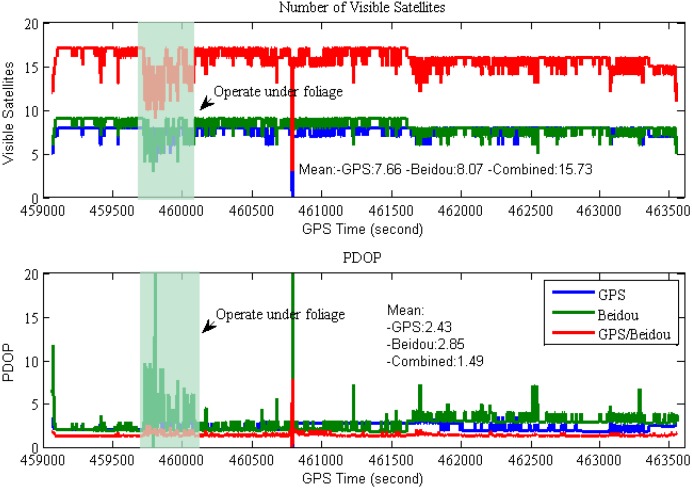
Satellites visibility and PDOP during the field test (15° cut-off elevation).

The satellite visibility and PDOP variations are shown in Figure 6. From this figure, one can find the number of tracked GPS and BDS satellites at 15° cut-off elevation is larger than ten, except when the vehicle drove under dense foliage or signal lock-lose occurred, which implies the availability of high-precision solution will increase significantly when GPS and BDS are combined, and it shows the PDOPs of the combined GPS/BDS system are less than 2 most of the time, which indicates better positioning performance can be achieved for the combined system when compared with that of single system.

During our test, more BDS satellites are visible compared to the GPS one during most of the time period, however the BDS PDOPs are slightly worse than those of GPS-only case due to the current BDS geometry configuration deficiency. The mean EDOP, NDOP, HDOP, VDOP and PDOP for the GPS/BDS combined system are also illustrated in Table 2, it clearly shows that the geometric strength is significantly improved for the combined system. The mean NDOP and VDOP are obviously worse than EDOP for both GPS-only and BDS-only case, besides the BeiDou’s NDOPs are obviously larger than those of GPS-only, whereas the EDOPs and VDOPs are comparable to those of GPS-only case.

**Table 2:** Mean DOP for the GPS/BDS combined system during the field test (15° cut-off elevation).

Configuration	EDOP	NDOP	HDOP	VDOP	PDOP
GPS	0.66	1.03	1.23	2.10	2.43
BDS	0.76	1.76	1.96	2.06	2.85
GPS/BDS	0.47	0.62	0.80	1.27	1.49

**Figure 7:**
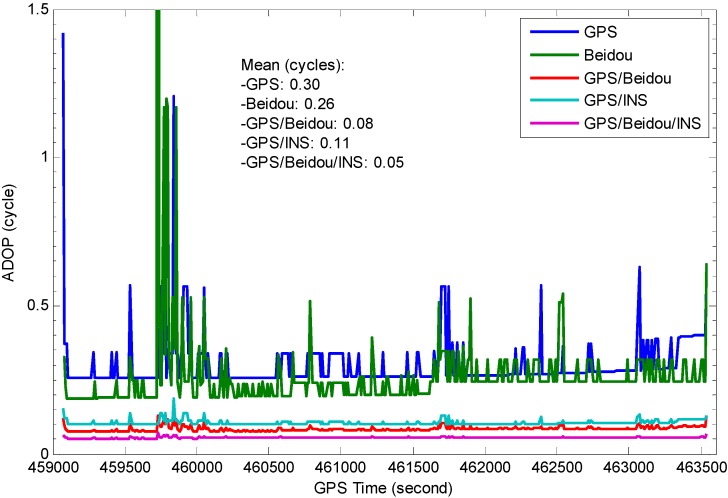
Impact of system configuration on ADOP (INS position STD 0.1 m, 15° cut-off elevation).

In Figure 7, the single epoch ADOP time-series over the navigation period for L1 GPS, B1 BDS, L1 GPS + B1 BDS, L1 GPS + INS, L1 GPS + B1 BDS + INS are demonstrated, the code STD are taken 0.37 and 0.35 m for GPS and BDS respectively, the phase STD is 0.01 cycles and INS position STD is set as 0.1 m for each axis. The figure shows the ADOPs of B1 BDS are slightly smaller than those of L1 GPS which is influenced by the number of visible satellites, thus better AR performance is expected to be achieved for BDS-only system, and it is obviously that ADOPs fluctuate strongly with poor satellite coverage, which indicates the performance of single-frequency instantaneous AR process is limited due to large ADOPs for the single system. The combined L1 GPS and B1 BDS obviously outperform single system and the ADOP time series are more stable, the instantaneous success-rate performance of the combined system is significantly improved. The figure also shows the ADOP time series of L1 GPS/INS combined system has an almost identical behavior as that of L1 GPS and B1 BDS combined system, this is due to precise priori position information provided by INS. As expected, the GPS/BDS/INS combined system achieves the best performance, the ADOP values are below 0.1 cycles which indicates high AR success-rate performance can be achieved.

**Figure 8:**
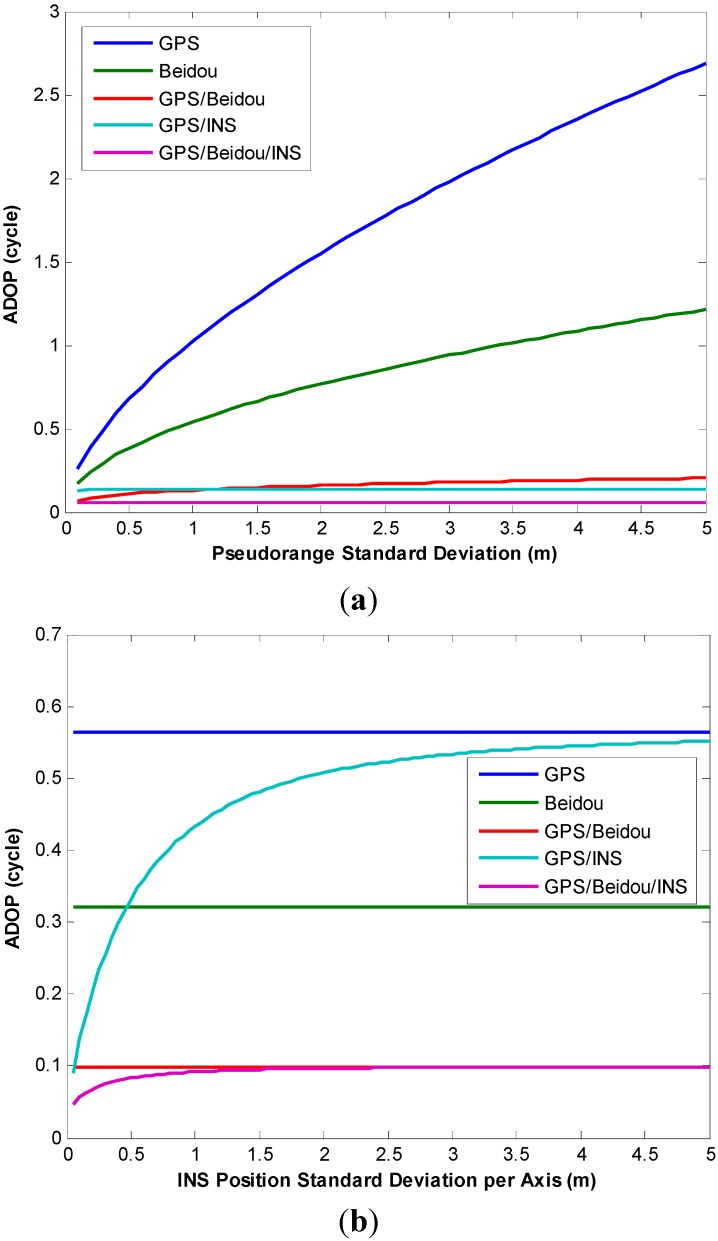
(**a**) Effect of pseudorange accuracy on ADOP; (**b**) Effect of INS position accuracy on ADOP.

More analyses were conducted to study the influence of pseudorange accuracy and INS position accuracy on AR performance, Figure 8a depicts the effect of pseudorange accuracy on ADOP at epoch 459,910 s, the effect of INS position accuracy on ADOP is shown in Figure 8b. It can be seen from Figure 8a that the ADOPs of a single system vary significantly depending on the magnitude of pseudorange errors. However, those values become significantly smaller for the integrated GPS/BDS system which means higher success rates can be obtained. As expected, the addition of INS measurements significantly improves the AR performance. On the other hand, INS predicted position errors grow during the GNSS outages, and the quality of INS bridging heavily depends on the outages duration and the quality of IMU sensor. As can be seen in Figure 8b, the ADOPs of the INS-aided system are still less than those of a single GNSS system when the INS position STD varies from 5 cm to 5 m, however the ADOPs of this GPS/BDS/INS combined system have a similar behavior as that of GPS/BDS combined system after INS position STD exceeds 2 m, it indicates the benefits to AR from INS-aided system becomes negligible after long GPS/BDS outages.

Since most of common error sources between reference and rover stations can be eliminated by the DD technique for the short-baseline configuration, the residual errors are mainly composed of multipath errors. In this paper, the twenty seven states GPS/BDS/INS TC EKF was implemented, the process noise parameters were turned by using the available sensor specifications and the results extracted from Allan variance (AV) analysis. The IMU sensor errors are estimated by the EKF, and raw INS measurements are then calibrated with the optimal estimates which will improve the INS navigation performance during GNSS outages.

**Figure 9:**
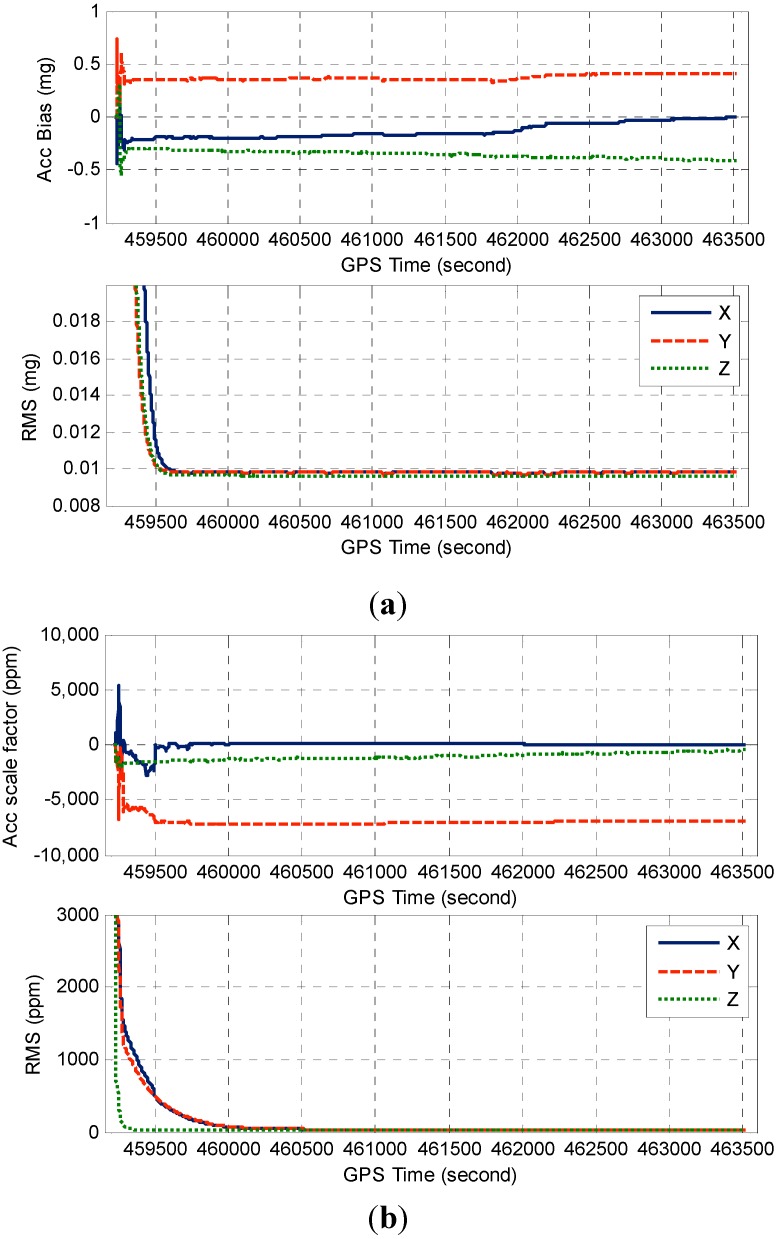
(**a**) Estimated accelerometer biases and RMS; (**b**) Estimated accelerometer scale factors and RMS.

**Figure 10:**
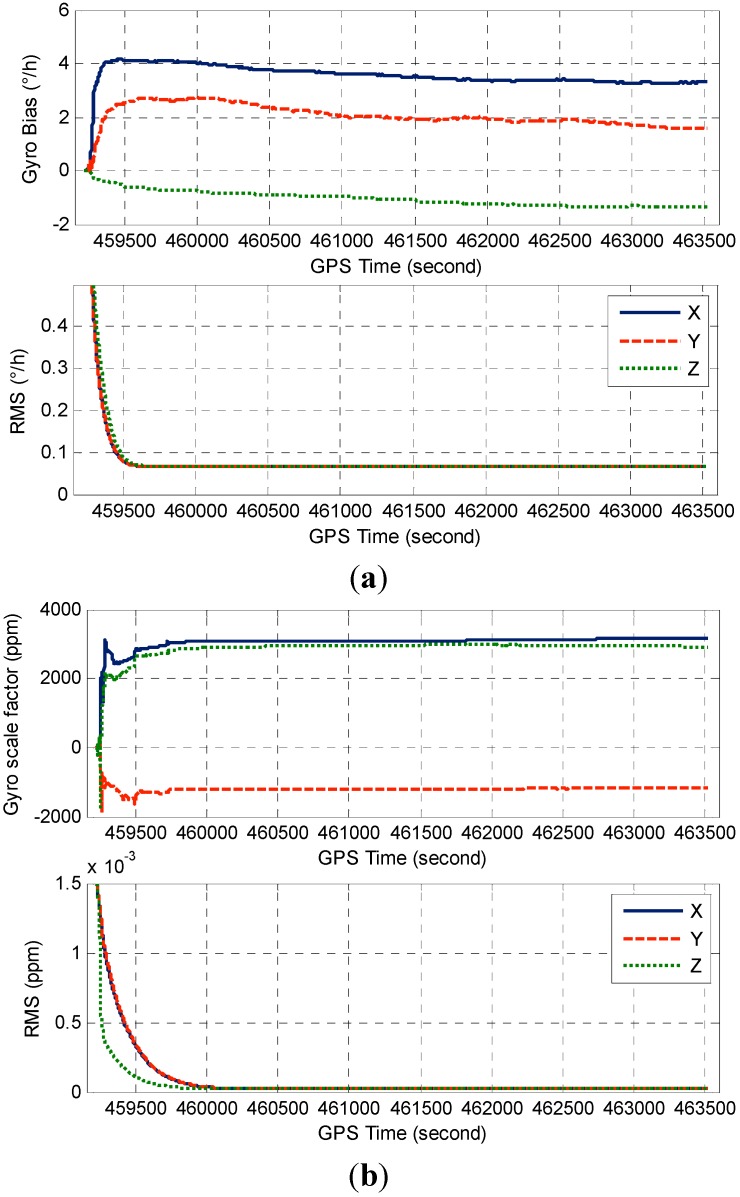
(**a**) Estimated gyro biases and RMS; (**b**) Estimated gyro scale factors and RMS.

Figure 9a illustrates the accelerometer bias and its RMS derived from the TC filter covariance matrix, Figure 9b displays the corresponding accelerometer scale factor estimates and the RMS. The gyro error estimates and the corresponding RMS are shown in Figure 10. As can be seen from Figure 9 and Figure 10, the IMU sensor error estimates quickly converge to stable values after the initial transition period, and the scale factor estimates somehow converge slower than those of bias estimates due to its weak observability.

The ambiguity resolution is a critical process for high accuracy positioning applications. In this research, six experiment schemes were designed to investigate the AR performance of dual-frequency single-epoch kinematic positioning: scheme 1—GPS, scheme 2—BDS, scheme 3—GPS/BDS, scheme 4—TC GPS/INS, scheme 5—TC BDS/INS, scheme 6—TC GPS/BDS/INS. Here we have evaluated the ambiguity fixing rate of single epoch AR at different elevation mask angles which represent different observation conditions, a total of 4200 epochs data have been processed, the fixed solution was validated by using the ratio test with the thresholds three.

As shown in Table 3, the fix rates of the individual GNSS system are less than 95% under all conditions, and the fix rates of BDS are comparable to those of GPS when the cut-off elevation angle is lower than 25°, with higher cut-off elevations, the number of tracked GPS satellites is on the decrease and GPS AR performance degrades dramatically, however, the degradation of BDS AR performance is not significant due to the special constellation of BDS. The integration of GPS and BDS has achieved a significant improvement in the availability and reliability, the fixed rates of single-epoch GPS/BDS AR are larger than those of single system under high-elevation conditions, however AR performance degrades under low-elevation conditions because of the presence of low-elevation multipath interference, the explanation lies in bias in the estimates induced by biased observations, and the incorrect fixed solution is then rejected by the validation process even though success rates are very high based on the formal analysis. As the cut-off elevation angle increase to 20°, the impact of multipath weakens and fixed rates increase.

**Table 3:** Ambiguity fixing rate of the dynamic test for 10°–40° cut-off elevations, using different data process strategies.

	Fix Rate (%)	*Cut-Off Elevation (*°*)*						
Configuration		10	15	20	25	30	35	40
GPS		84.05	86.84	92.90	92.00	74.59	44.26	31.07
BDS		86.73	89.52	91.77	92.59	88.57	80.55	80.55
GPS/BDS		75.27	82.94	93.68	94.79	96.21	96.90	96.59
TC GPS/INS		98.50	98.17	98.67	98.76	98.86	99.69	86.38
TC BDS/INS		93.72	96.24	97.60	97.84	97.95	97.41	97.41
TC GPS/BDS/INS		95.93	96.93	98.90	99.14	99.50	99.62	99.57
TC GPS/INS (ad)		98.79	98.95	99.29	99.55	99.26	99.66	86.40
TC BDS/INS (ad)		95.29	96.26	98.55	98.26	98.90	98.76	98.76
TC GPS/BDS/INS (ad)		96.48	96.95	98.93	99.21	99.57	99.83	99.86

**Figure 11:**
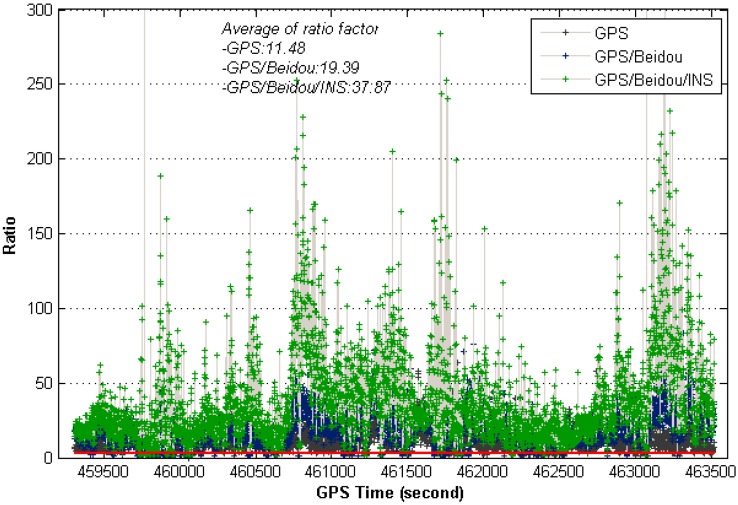
Ratio values of instantaneous AR for different system configurations (20° cut-off elevation).

However, the most impressive thing in the results is that the fix ratio is significantly improved with the addition of INS to GNSS. Figure 11 shows the ratio test results by the improved INS aiding strategy at 20° cut-off elevation angle, instances where the ratio-value get below three is indicated by the red line. The figure clearly shows ratio values from INS aiding strategy are significantly larger than GNSS-only ones, which means the proposed INS aiding strategy is effective to enhance the fixing probability.

In order to mitigate the multipath impact on AR performance, the adaptive fading memory stochastic modeling strategy is adopted, the fading factor is taken 0.85, and the corresponding results are also listed in Table 3 for comparison. It clearly shows the proposed strategy is effective for improving the AR performance, the fix ratio is improved relative to the strategy using the *a priori* elevation-dependent weighting scheme, and a slightly larger improvement for TC BDS/INS has been achieved, indicating a more accurate stochastic model is beneficial for AR. Besides, the multipath effect is suppressed due to the *a priori* INS constraint which indicates a better AR performance can be achieved at lower cut-off elevations. We can also see TC GPS/BDS/INS system brings much advantage for AR at higher cut-off elevations, the fixing rates increase to 99% with cut-off elevations up to 25°.We also recognize AR performance of GPS is slightly better than BDS with the aid of INS.

**Figure 12:**
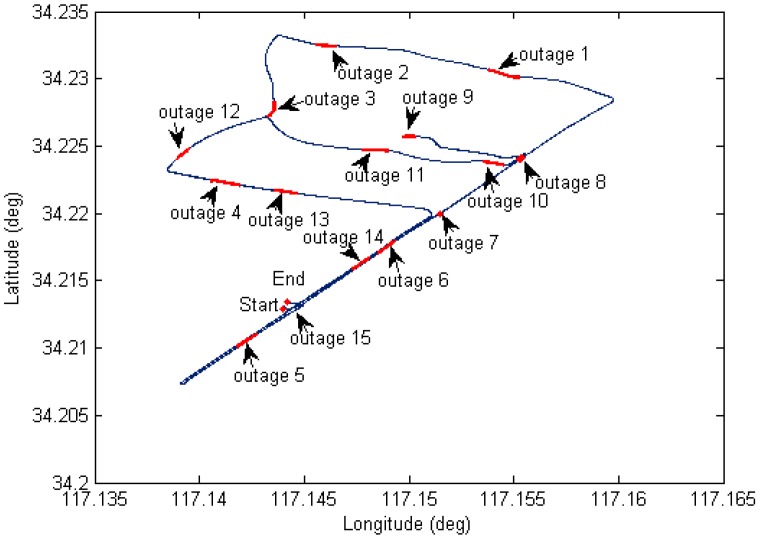
Ground track of test vehicle with simulated GNSS outages.

**Figure 13:**
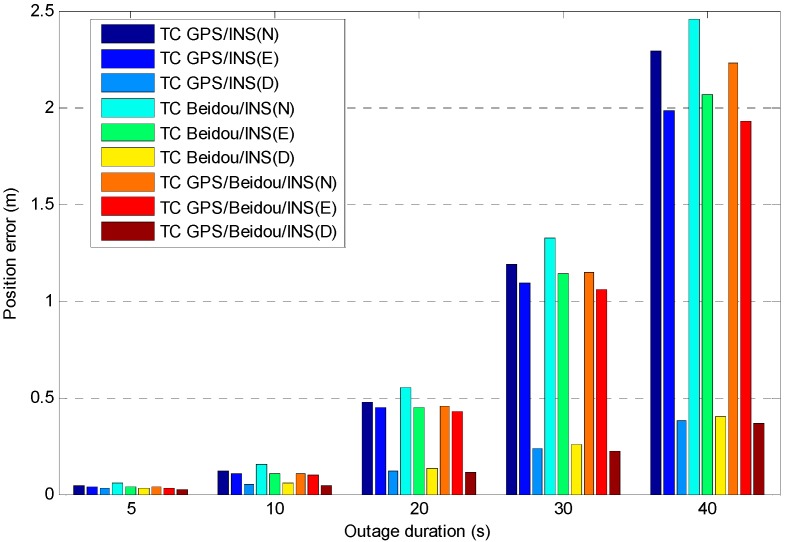
Average of the maximum position error *vs.* outage duration for different system configurations.

For high accuracy kinematic positioning applications, the capability to provide a robust and continuous solution is critical to maintain the positioning performance. In harsh navigation environments, e.g., while driving through tunnels or along unban canyons, the satellite availability is reduced or the solution is even unavailable, so we expect to achieve fast ambiguity recovery over long outages with the help of INS. In order to evaluate INS-only position performance during GNSS outages, we have designed fifteen complete GNSS outages under different vehicle dynamics (see Figure 12), five different outage durations for each outage were considered (5, 10, 20, 30 and 40 s). We used the maximum error as the measure of positioning performance due to the fact it typically occurs at the end of the outage, and the combined GPS/BDS fixed solution was used as a reference, any abrupt departure point in the reference trajectory has been removed. The average maximum error of each position component for the fifteen outages is shown in Figure 13, where it can be seen the INS position errors stay at a reasonable level after the calibration processes. The error growth behaves with a similar tendency for the three different TC configurations, with a slightly better performance obtained from TC GPS/BDS/INS integration. The positioning performance for the easting and northing component is worse than the height component which is mainly caused by the poorer heading estimates. 

**Figure 14:**
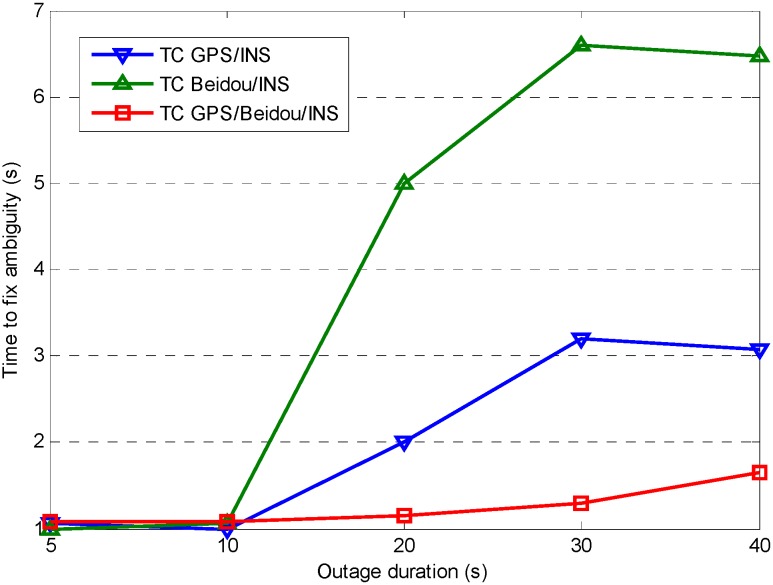
Time to fix ambiguity after different outage durations for different system configurations.

Figure 14 shows the time-to-fix ambiguities based on the analysis on the 15 completed outages. The results indicate AR performance is similar for TC GPS/INS, TC BDS/INS and TC GPS/BDS/INS over a short outage period (outage duration of less than 10 s), however AR performance of TC GPS/BDS/INS configuration shows a significant improvement after a long signal outage, as it can provide instantaneous fixed solutions even in the case of a 40 s outage. This has direct benefits to system reliability and availability.

**Figure 15:**
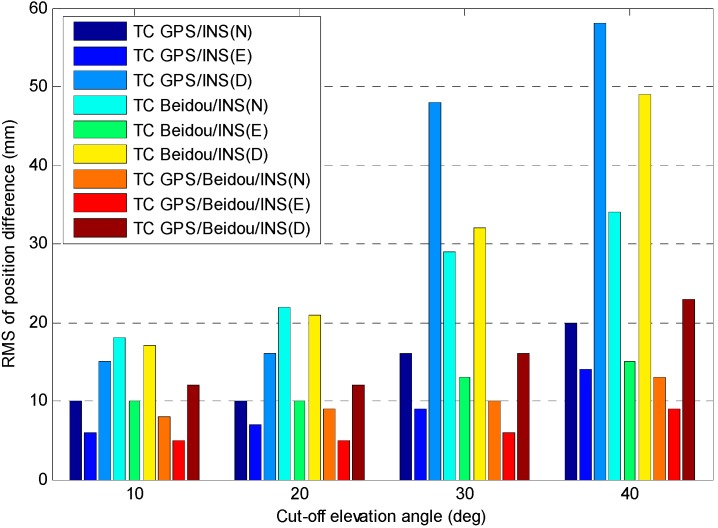
RMS of position difference *vs.* cut-off elevation angle for different system configurations.

The AR results in Table 3 are very promising, however the positioning performance is related to satellite geometry and INS positioning quality. We have evaluated the positioning performance of combined system at different cut-off elevations, where the reference solution is GPS/BDS fixed solution. Figure 15 presents the RMS of the position difference for different system configurations. 

It shows that the positioning performance of TC GPS/INS and TC BDS/INS degrades significantly as the cut-off elevations increase, however it only shows a slight degradation in positioning accuracy for the TC GPS/BDS/INS system, with the cut-off elevation angle of 40°, the RMS of position difference is 13, 9 and 23 mm for TC GPS/BDS/INS system in N, E and D components, respectively, this becomes particularly beneficial when positioning in constrained environments, e.g., in urban canyons or when low-elevation multipath interference is dominant. The figure also shows the position accuracy for TC GPS/INS is superior to the TC BDS/INS one, it can also be seen that the accuracy in N and D component are obviously worse than that in E component, especially for BDS, and such results are consistent with the DOP values as illustrated in Table 2.

**Figure 16:**
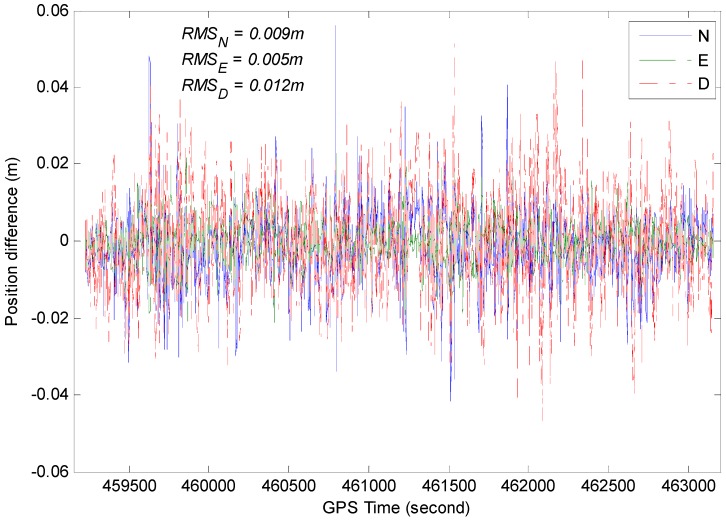
Position differences between GPS/BDS/INS integration solution and GPS/BDS solution (20° cut-off elevation).

A comparison between GPS/BDS/INS integration solution and GPS/BDS fixed solution at 20° cut-off elevation is provided in Figure 16. The difference stays below 4 cm most of the time for each component, and occasionally reaches 6 cm, which may be caused by vehicle dynamics.

**Figure 17:**
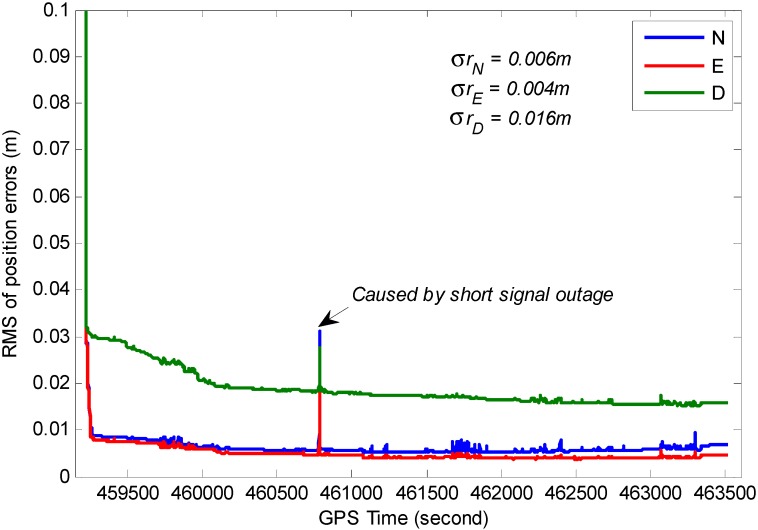
RMS of the estimated position errors.

**Figure 18:**
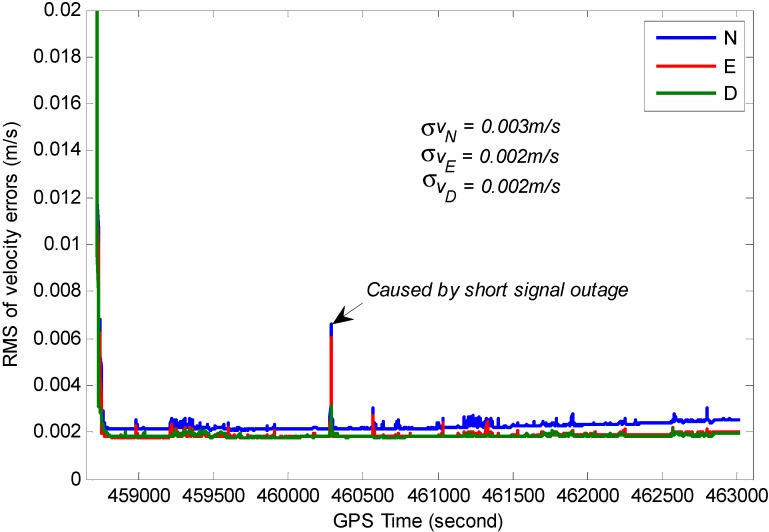
RMS of the estimated velocity errors.

**Figure 19:**
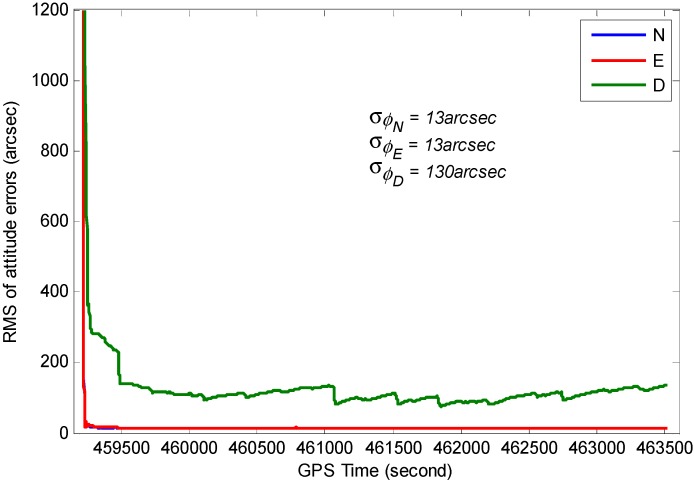
RMS of the estimated attitude errors.

The RMS for position, velocity and attitude of the TC GPS/BDS/INS system based on variance analysis are illustrated in Figure 17, Figure 18 and Figure 19. From these figures, we can see the estimated position accuracy for horizontal components is better than 1 cm, whereas vertical component position accuracy is generally better than 2 cm. It can be seen that an abrupt change occurs in the RMS time-series, this is caused by GNSS signal lock-loss. The estimated velocity accuracy is generally better than 3 mm/s. The achievable attitude accuracy for pitch and roll component reach 13 arc seconds, while a much poorer performance can be obtained for the heading component for the test.

**Figure 20:**
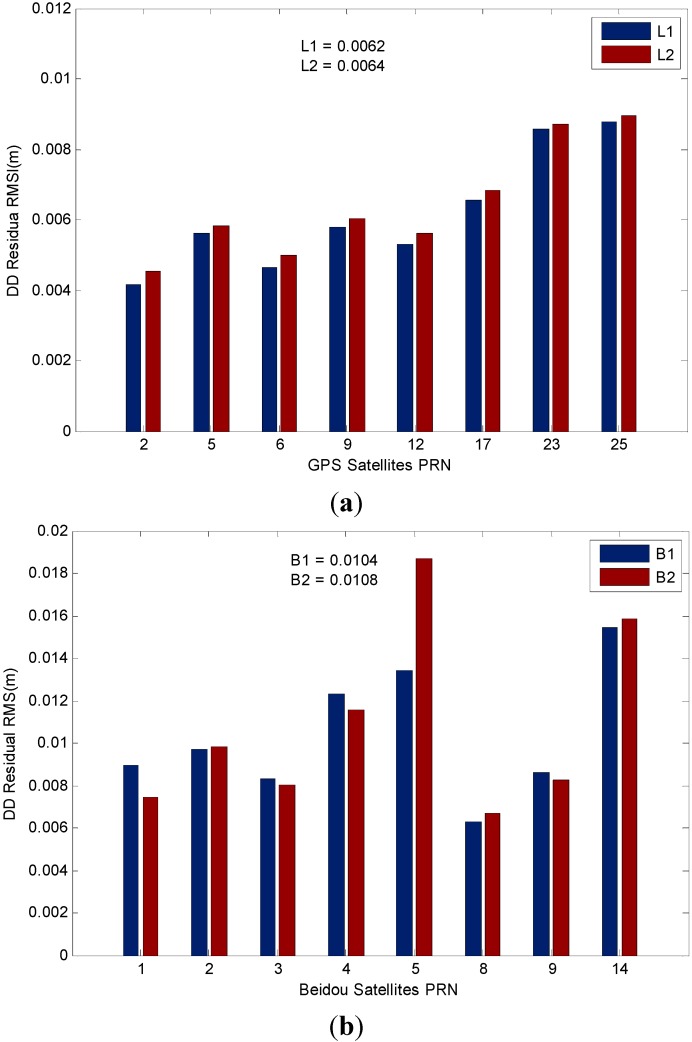
(**a**) RMS values of GPS DD residual; (**b**) RMS values of BDS DD residual.

We also illustrate the positioning performance of the GPS and BDS system in the GPS/BDS/INS tightly integrated navigation system by analyzing the residual of the measurements. The RMS value statistics of L1 (B1) carrier-phase residual when ambiguities are fixed are illustrated in Figure 20. The average RMS values are 6.2 and 6.4 mm for GPS L1 and L2 carrier-phase, respectively. The low elevation angle GPS satellites (G23, G25) have larger residuals which may be affected by multipath errors. For BDS satellite system, the RMS values for B1 and B2 carrier phase are up to 10.4 and 10.8 mm, respectively, it is slightly worse than the GPS ones, we can also find that residuals are varied with the type of satellites, among which the IGSO satellites have better performance in this study, the only MEO satellite C14 has the worst performance, and B2 residuals of GEO satellite C05 are apparently larger than the B1 residual due to the measurement fluctuations.

## 6. Conclusions

In this contribution, we have developed a tightly coupled system for the integration of GPS, BDS and MEMS-based inertial system to improve the estimation accuracy and reliability in positioning. The tightly integration based EKF was implemented by directly fusing ambiguity fixed double-difference (DD) carrier phase measurements with the INS predicted pseudoranges to estimate the error states. The algorithm concerned with high accuracy positioning was addressed, which includes single epoch AR aided by INS. We have designed and performed a vehicular test to verify the positioning performance of the integration system. The highlights of this work are as follows:

(1) Compared with the single system, the availability and reliability of the combined GPS/BDS system are dramatically improved, and the satellite visibility is still maintained in constrained environments (e.g., in urban canyons).
(2) We have analyzed the AR performance for different system configurations under different cut-off elevation conditions, the results indicate the single epoch ambiguity fixing rates of the TC GPS/BDS/INS system are significantly improved as compared to that of GNSS-only or single satellite navigation system integrated strategy, especially for high cut-off elevations. The results are very promising and indicate the increasing applicability for high accuracy positioning in constrained environments. The AR performance of such an integrated system, in low elevation multipath environments, is improved by employing the adaptive fading memory stochastic modeling strategy.
(3) In addition, we have examined the INS bridging performance for the low cost MEMS IMU during GNSS outages, and the results indicate the AR performance is similar for TC GPS/INS, TC BDS/INS and TC GPS/BDS/INS over a short outage period (outages duration less than 10 s), however there has been a significant improvement in AR performance of TC GPS/BDS/INS systems in the case of long outages, where the bridging allows instantaneous ambiguity recovery after 40 s outage in the test.
(4) Beside the analysis on AR performance, we have investigated the positioning performance of the integrated system under different cut-off elevation conditions, and the results indicate the positioning performance of TC GPS/BDS/INS system outperforms that of TC GPS/INS and TC BDS/INS, as the TC GPS/BDS/INS integration system achieves a few centimeters accuracy in positioning based on the comparison analysis and covariance analysis even in harsh environments, thus we can see the advantage for positioning at high cut-off elevations that the combined GPS/BDS brings.
(5) The analysis on the DD carrier-phase residuals indicates that BeiDou phase residuals are larger than the GPS ones, and the BeiDou phase residuals are satellite dependent, among which the IGSO satellites shows a better performance, while GPS the residuals vary with elevation.

Future work will concentrate on an integration algorithm for a multi-GNSS system (GPS, BeiDou, GLONASS and Galileo), quality control algorithm of integration system for high-accuracy positioning, and further tests for long-baseline positioning will be carried out in the future.
